# Supplementary material for: Adaptive and non-adaptive gene expression responses in prostate cancer during androgen deprivation
Source: PLoS One. 2023 Feb 21;18(2):e0281645. doi: 10.1371/journal.pone.0281645 (PMC9942993; doi:10.1371/journal.pone.0281645)
Supplement: S1 Fig — Analysis of androgen receptor (AR) protein levels by Western blotting with relative density values. Whole Western blots are shown. All measured densities were corrected with corresponding Beta-actin. For both Western blots separately the density of control was set to 1 and the densities of the other lanes were calculated relative to that. More than 2-fold differences were considered to be markedly changed. (PDF) [file pone.0281645.s001.pdf]

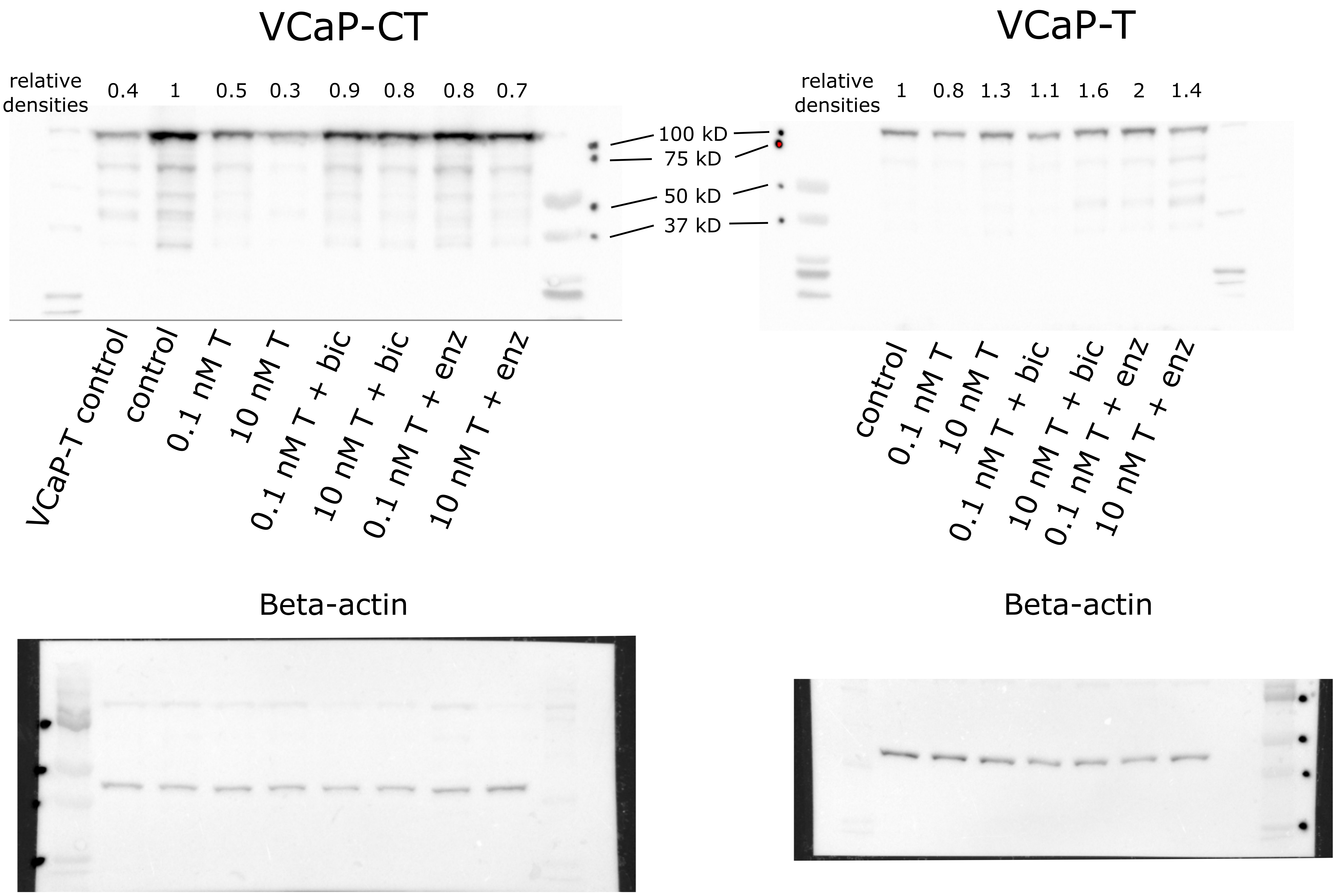

Figure S1: Analysis of androgen receptor (AR) protein levels by Western blotting with relative density values. Whole Western blots are shown. All measured densities were corrected with corresponding Beta-actin. For both Western blots separately the density of control was set to 1 and the densities of the other lanes were calculated relative to that. More than 2-fold differences were considered to be markedly changed.
