# Supplementary material for: Adaptive and non-adaptive gene expression responses in prostate cancer during androgen deprivation
Source: PLoS One. 2023 Feb 21;18(2):e0281645. doi: 10.1371/journal.pone.0281645 (PMC9942993; doi:10.1371/journal.pone.0281645)

For all images: Chemiluminescence of immunoreactive band was detected by using ChemiDocTM XRS+ -equipmet (Bio-Rad, Hercules CA, USA).

Androgen receptor VCaP-CT

Fig 2 A: First blot from the top, S1 Fig: Upper left blot

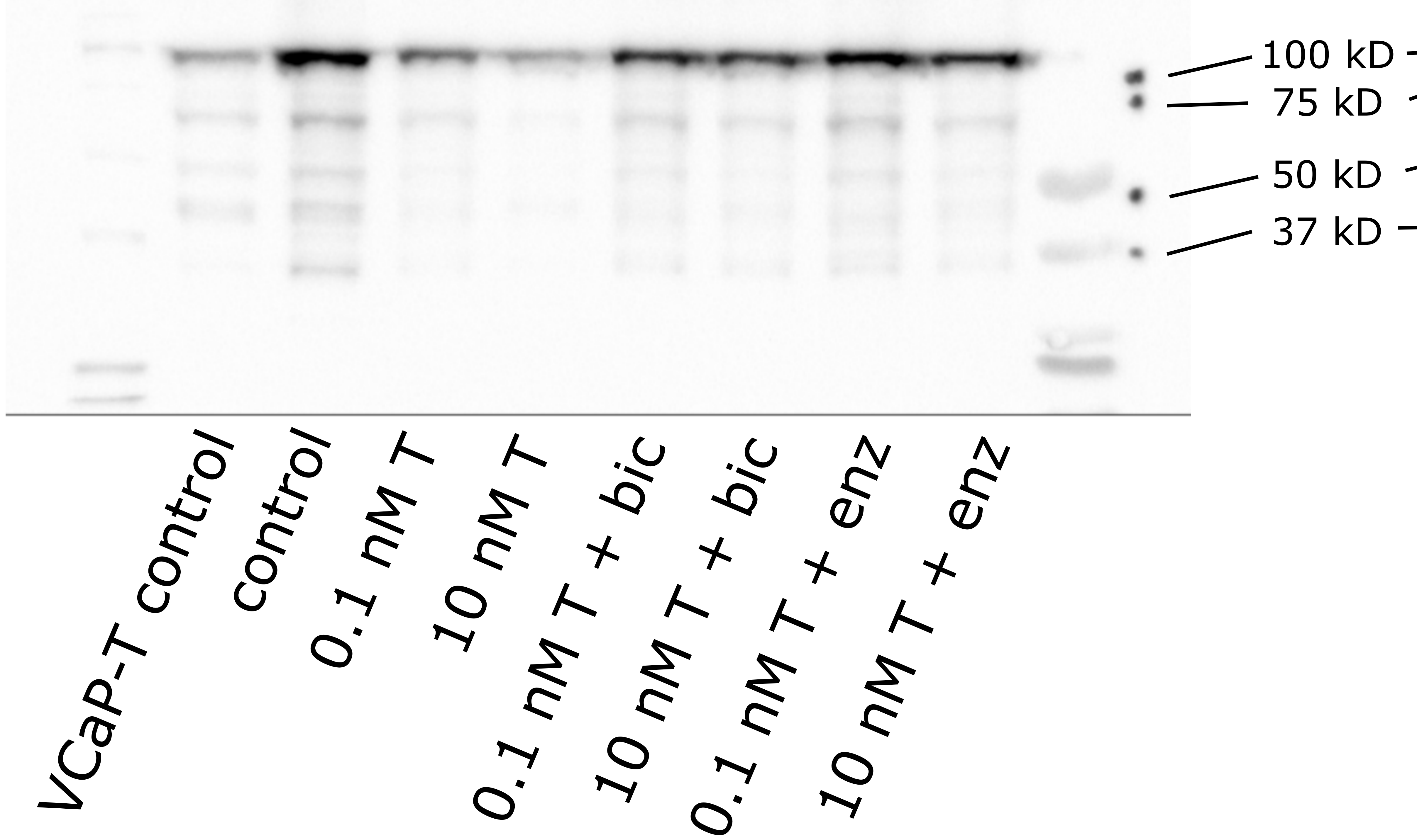

Androgen receptor VCaP-T

Fig 2 A: Third blot from the top, S1 Fig: Upper right blot

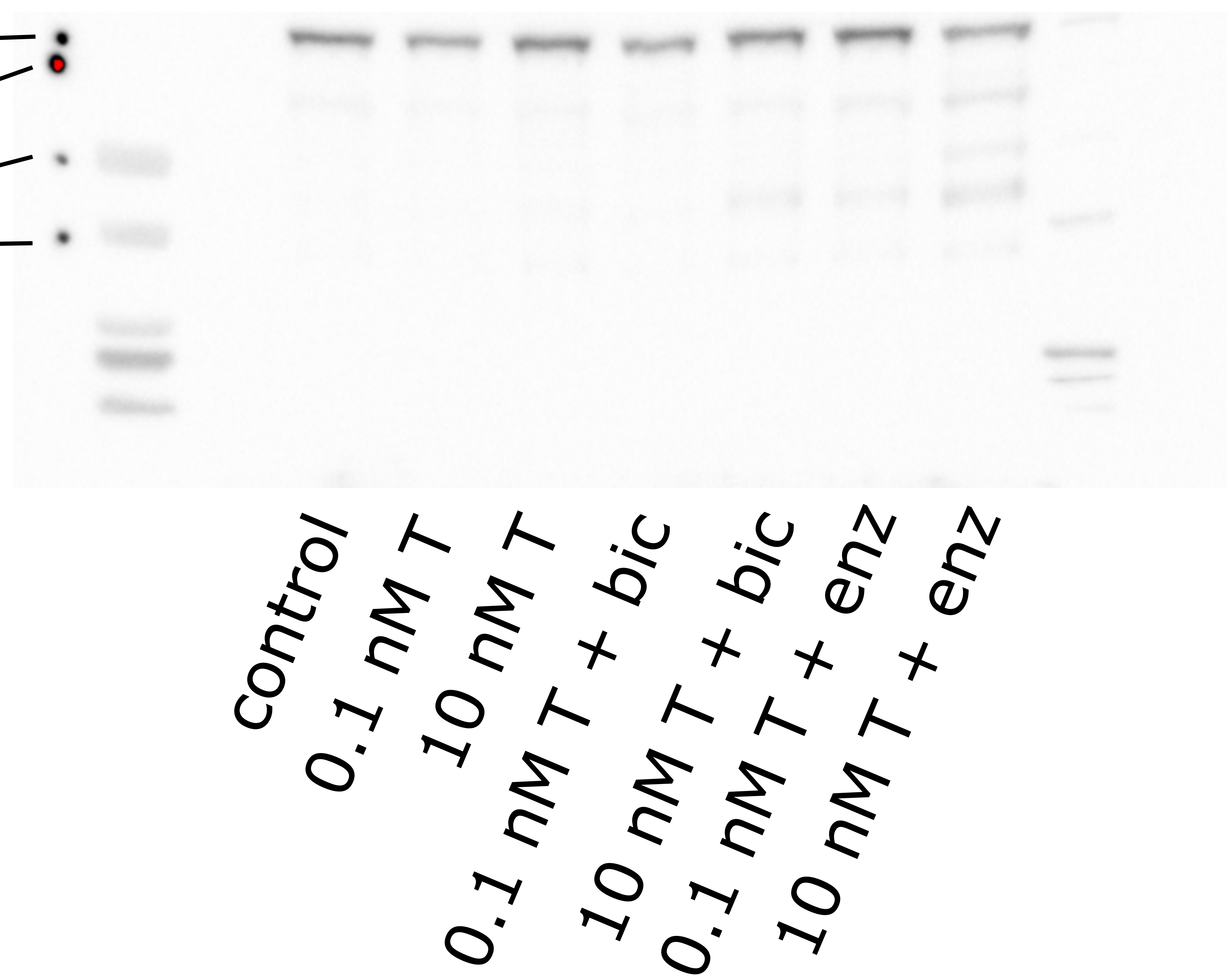

Beta-actin VCaP-CT

Fig 2 A: Second blot from the top, S1 Fig: Bottom left blot

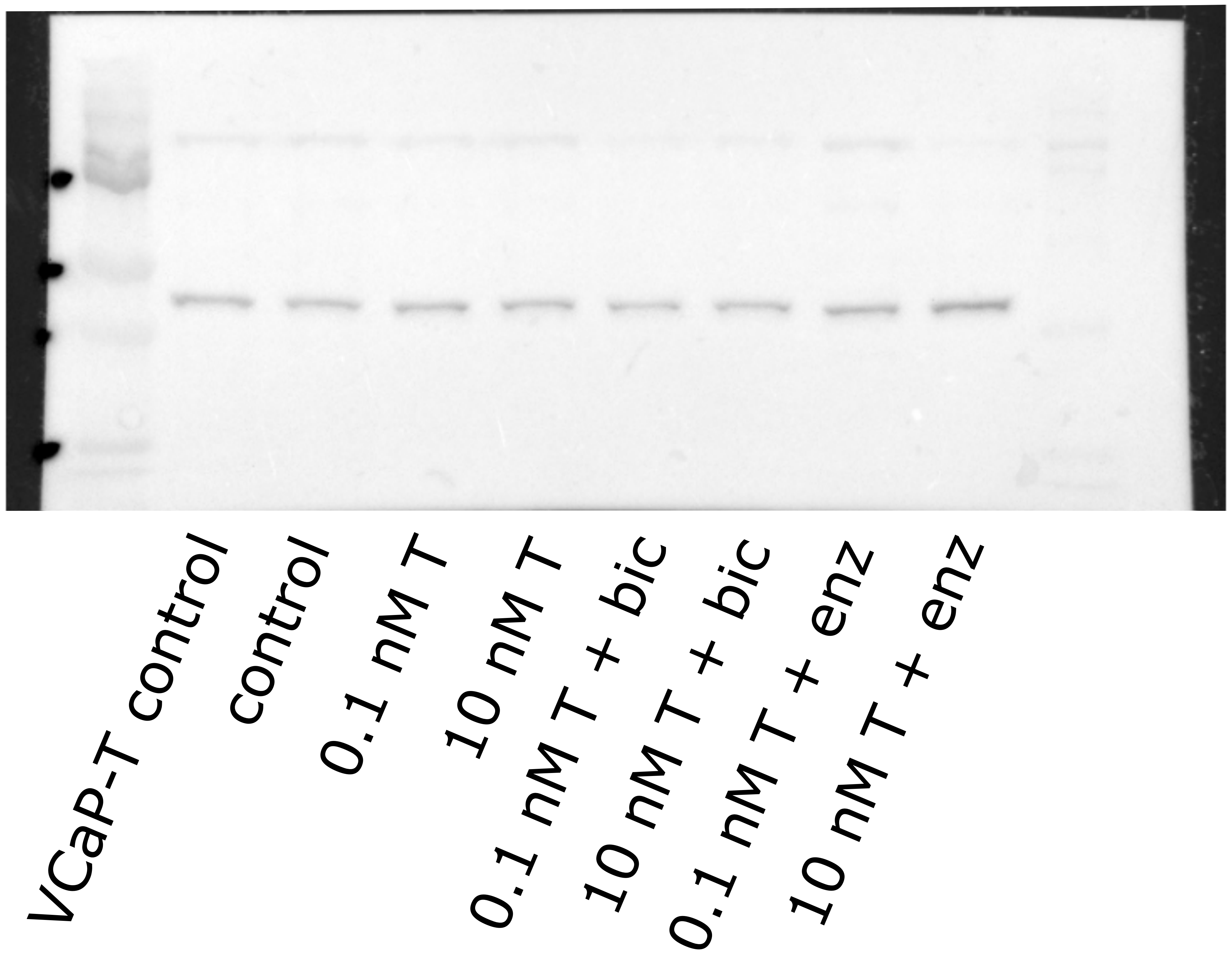

Beta-actin VCaP-T

Fig 2 A: Fourth blot from the top, S1 Fig: Bottom right blot

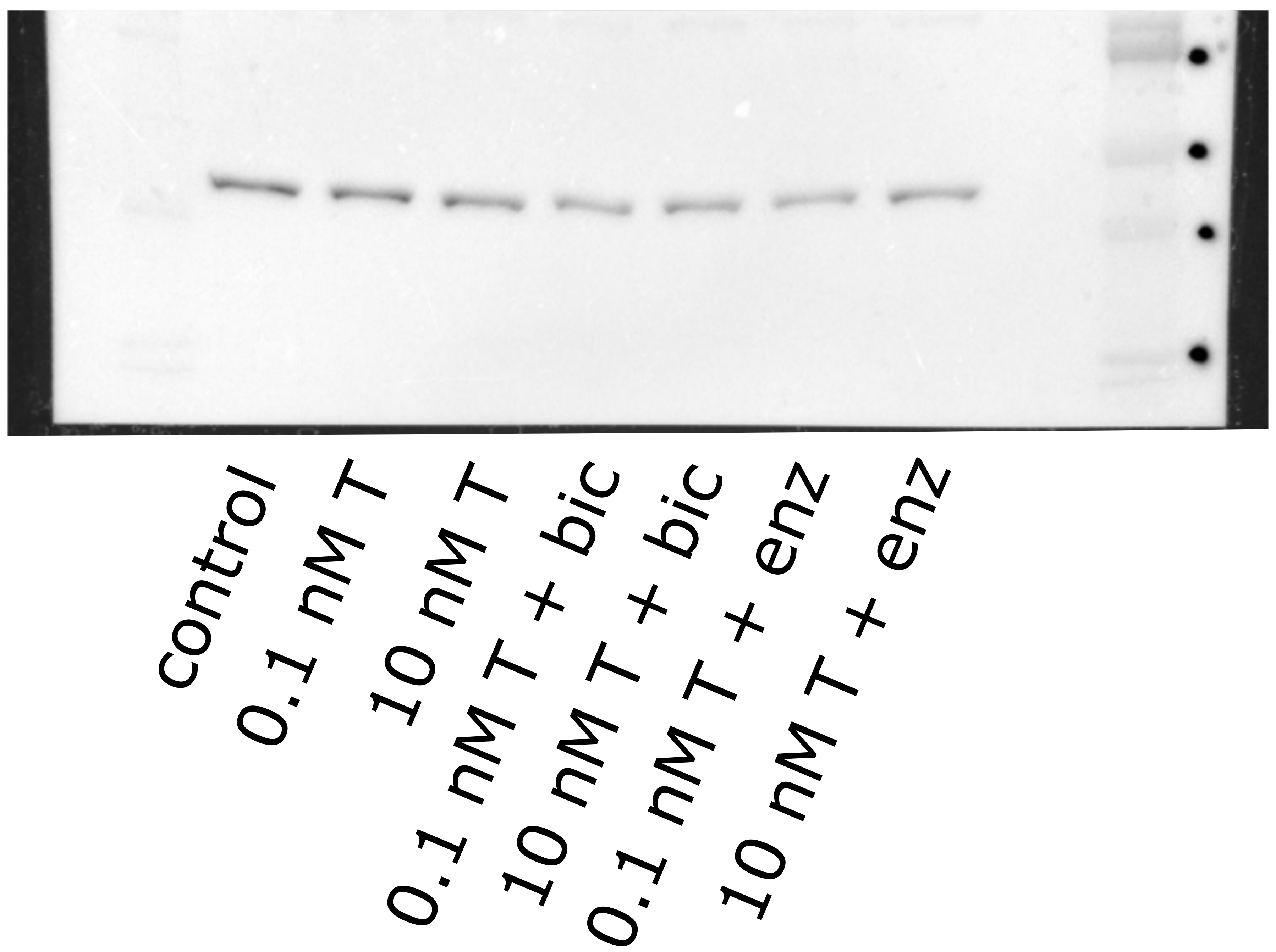

Supplement: S1 Raw images — (PDF) [file pone.0281645.s008.pdf]
